# Early-onset liver cancer in South America associates with low hepatitis B virus DNA burden

**DOI:** 10.1038/s41598-018-30229-8

**Published:** 2018-08-13

**Authors:** Agnès Marchio, Juan Pablo Cerapio, Eloy Ruiz, Luis Cano, Sandro Casavilca, Benoît Terris, Eric Deharo, Anne Dejean, Stéphane Bertani, Pascal Pineau

**Affiliations:** 1 Institut Pasteur, Unité “Organisation Nucléaire et Oncogenèse”, INSERM U993 Paris, France; 2 Sorbonne Universités, UPMC Université Paris 06, Paris, France; 3 Instituto Nacional de Enfermedades Neoplásicas, Departamento de Cirugía en Abdomen, Lima, Peru; 4 Université de Rennes 1, INSERM, CNRS, U 1241 NUMECAN Rennes, France; 5 Instituto Nacional de Enfermedades Neoplásicas, Departamento de Patologia, Banco de Tejidos Tumorales, Lima, Peru; 6 Assistance Publique-Hôpitaux de Paris, Hôpital Cochin, Service d’Anatomie et Cytologie Pathologiques, Paris, France; 7 Université de Toulouse, UPS, UMR152 PHARMADEV, Université Toulouse 3, Toulouse, France

## Abstract

In Peru, hepatocellular carcinoma (HCC) arises in young non-cirrhotic patients. Hepatitis B virus (HBV) is suspected to be the prominent etiological agent. We thus performed a comprehensive molecular study of HBV infection in 65 Peruvian HCC patients. Only 51% were considered as persistently infected at the onset. HBV DNA was found by PCR in the tumor and/or matched non-tumor liver tissues in more than 80% of cases (n = 53/65). HBV DNA was significantly more abundant in livers of younger patients than in those of the older ones. We consistently observed low viral DNA burden (0.1–6.5 copies for 100 cells), with viral genomes in younger patients displaying higher proportion of mutations at di-pyrimidines (TpT and CpC, P = 0.006). A drastic activation of multiple DNA repair pathways in tumors of younger patients was observed. Our observations clearly challenge the current vision that associates high HBV DNA load with earlier tumor development. We concluded that in Peru, and maybe in other populations with Americas’ indigenous ancestry, HBV-associated liver tumorigenesis might differ significantly from that generally observed in the rest of the world. Procedures used to screen for HCC development in subjects at risk should be adapted to the local situation.

## Introduction

In the past decades Hepatocellular carcinoma (HCC) has become a major cause of death by cancer worldwide, especially in low- and middle-income countries^1,2^. However, HCC is not considered in South America as a prominent malignancy when compared to gastric, lung, or even cervix cancers^2^. A recent review of the South American literature indicated that patients from the region that develop HCC are typically hepatitis C virus (HCV)-infected males over 60 years^3^. Yet, at odds with the general South American pattern, the most important risk factor of HCC worldwide remains chronic hepatitis B^4^.

Hepatitis B virus (HBV) is known to be instrumental in liver carcinogenesis either by DNA integration in host genes, oncoproteins production, or through an abundant viral replication presumably stimulating immune pathogenesis, notably in East Asian patients^5^. Most of South America is considered a zone with low to intermediate endemicity for HBV, with the Amazon rainforest basin that covers eastern parts of Peru as a notable exception^6^. In this sparsely populated region, chronic infection with HBV is highly prevalent in remote indigenous communities^7^.

Significant variations of HBV seroprevalence are observed in Peru, with a decreasing East-West gradient from the Amazon rainforest to the Pacific coastal areas^8^. Between these two poles stands the Andean mountains that shelter dispersed communities displaying occasionally very high HBV seroprevalence. These populations, primarily found in the Peruvian southern-central Andean areas (i.e. regions of Apurimac, Ayacucho, and Cusco), exhibit very high rates (>10%) of HBV surface antigen (HBsAg) carriage^8^. In these communities and in Peru in general, we reported a bimodal age-specific distribution of HCC cases with two peaks of incidence around 25 and 64 years^9^. A similar situation was described decades ago in Alaska, where HCC affected younger members of the Yupik native group. Remarkably, native Alaskans developing HCC at younger age were generally infected with subtype F1 of HBV, the very same viral clade that infects Peruvian patients^10,11^. The role played by genetic susceptibility in these early neoplasia remains unknown; but regarding ethnicity, it should be noted that Peruvian citizens are presenting among the highest proportions of Americas’ indigenous ancestry in South America^12^. Recently, it appeared that HCC presentation, firstly described by our group in Peru, might be more widespread on the South American continent than at first sight^13^.

Little is known about the molecular epidemiology of HCC in South American patients. The patients included in the present series have already been submitted to mutation analysis to detect alterations affecting major tumor suppressors and oncogenes^14^. We showed that there were in fact some differences between younger and older Peruvian HCC patients regarding the rate of alterations affecting genes of the *Wnt* pathway (e.g. beta-catenin- and axin-1-encoding genes). However, these differences do not represent a systematic causal explanation for tumor process, as they were present only in subsets of patients (40% in younger individuals vs. 15% in older ones). We thus decided to conduct a comprehensive molecular analysis of HBV genomes present in a series of HCC and parent non-tumor liver (NTL) tissues of 65 Peruvian patients managed at the National Cancer Institute of Peru (INEN), in order to gain further insights into the mechanisms at work in South American liver tumors.

## Results

### Hepatitis B virus DNA prevalence

We conducted a molecular survey on viruses present in HCC and NTL matched pairs (HCC/NTLs) of 65 Peruvian patients, for whom we had both DNA and RNA at disposal. The clinical demography of the patients studied are displayed in Table 1. We used both nested- and quantitative (q) PCR methods to detect and quantify HBV, HCV, as well as hepatitis D virus (HDV) in HCC/NTL of the 65 patients. Two patients were infected with HCV subtype 1b, whereas a single patient was infected with HDV genotype 3. These three patients were HBV carriers as well.

**Table 1:** Baseline demographical and clinical features of the Peruvian HCC patients.

Feature	Mean value or percent (±s.d.)	Range or proportion
**Demography**		
Age (years)	41.6 ± 2.6	[6–81]
*Sex-ratio* (M:F)	1.5	39:26
Birthplace in southern central Andes	33.8%	22/65
**Viral serology**		
HBsAg(+)	50.7%	33/65
Anti-HBc(+) total	72.3%	47/65
Anti-HDV(+)	1.5%	1/65
Anti-HCV(+)	3%	2/65
Tumor features		
Tumor size (cm-diameter)	14.4 ± 0.7	[5–30]
Number of liver segments involved	3.5 ± 0.2	[1–8]
Poorly differentiated	21.5%	14/65
Multi-nodular	27.6%	18/65
**Liver features**		
Cirrhosis	3%	2/65
Fibrosis	9.2%	6/65
Inflammation	26.1%	17/65
Steatosis	12.3%	8/65
Dysplasia	1.5%	1/65
Healthy liver	56.9	37/65
**Blood test**		
AFP (ng/mL)	1.3E + 05 ± 2.8E + 04	[1–8E + 05]
ALT (U/L)	61.7 ± 8.9	[8–330]
AST (U/L)	103.9 ± 13.5	[11–463]
ALP (U/L)	309.2 ± 33.3	[12–1,331]

Footnote: Percentages are expressed as a ratio of the 65 patients investigated for the considered parameter. Mean values are presented with ±standard deviation (s.d.). AFP, alpha-fetoprotein; ALP, alkaline phosphatase; ALT, alanine aminotransferase; AST, Aspartate aminotransferase; total Anti-HBc(+), antibody against hepatitis B core antigen; Anti-HCV, hepatitis C virus antibody; Anti-HDV, hepatitis D virus antibody.

**Figure 1:**
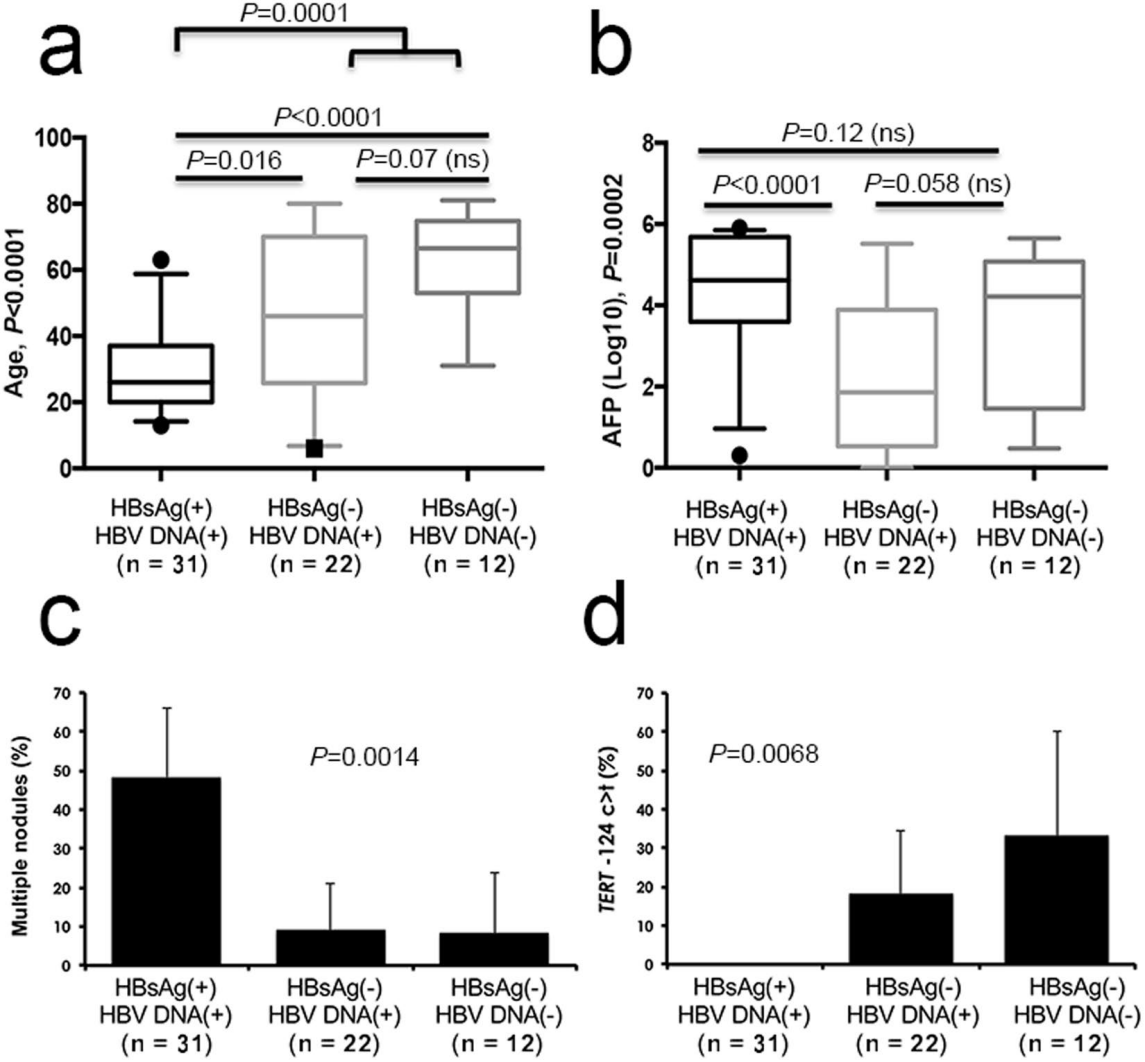
Viral status modulates clinico-biological features of Peruvian patients with HCC. (**a**,**b**) Box-and-whiskers plots. (**a**) Age (years) of patients with HCC according to the HBV markers detected. (**b**) AFP serum levels (ng/mL) measured according to the HBV markers detected. For age and AFP comparisons, P values were provided by Kruskal-Wallis test for global comparisons of the three groups while they were obtained by Mann-Whitney U test for the comparisons of groups against each other. (**c,d**) Bar charts. ns, not significant. (**c**) Tumor nodularity according to the HBV markers detected. (**d**) Level of mutations affecting *TERT*-encoding gene according to the HBV markers detected. (**c,d**) Error bars represent the standard deviation. P values were provided by Chi-square test.

HBV DNA amplification was positive in at least one specimen of the matched pair of liver tissues (tumor and/or non-tumor) in 81.5% of patients (n = 53). Such high proportion of positive cases is generally the hallmark of a series of patients living in countries with high endemicity of HBsAg seropositivity (≥8%)^4^. Patients positive for HBV DNA were significantly younger than negative ones (median ages 30 vs. 66.5; *P* < 0.0001). Remarkably, a notable fraction of HBV DNA(+) patients were HBsAg-negative (33.8%, n = 22). This latter pattern, corresponding to an occult HBV infection (OBI), was thus highly prevalent in this surgical series of HCC patients. Those with OBI were significantly older than patients with an overt infection with HBV (median ages 46 vs. 26; *P* = 0.016). The three infectious patterns (i.e. overt infection, OBI, and HBV DNA negative) were defining three age-related subsets (Fig. 1a). Patients HBsAg(+)/HBV DNA(+) also tended to display higher levels of AFP in serum (Fig. 1b). In addition, patients HBsAg(+)/HBV DNA(+) were more frequently affected with multiple nodule tumors at diagnosis (48.4% vs. 10%, OR = 8.4, 95%CI: 2.1–33.4; *P* = 0.001) (Fig. 1c). Finally, telomerase reverse transcriptase (*TERT*) −124 c > t mutation, considered the most frequent somatic mutation in HCC, was absent from tumors of HBsAg(+)/HBVDNA(+) patients (P = 0.0068) (Fig. 1d)^15^.

### Hepatitis B virus DNA loads in liver tissues

It has been shown that HCC and non-tumour livers of the same patient are not equivalent compartments regarding HBV replication and expression. Indeed, signs of HBV activity, such as amounts of cccDNA or viral RNA, are slightly easier to detect in the non-tumor liver than in the tumor itself where HBV DNA is often integrated in host cell genome under a form incompatible with replication^16^.

**Figure 2:**
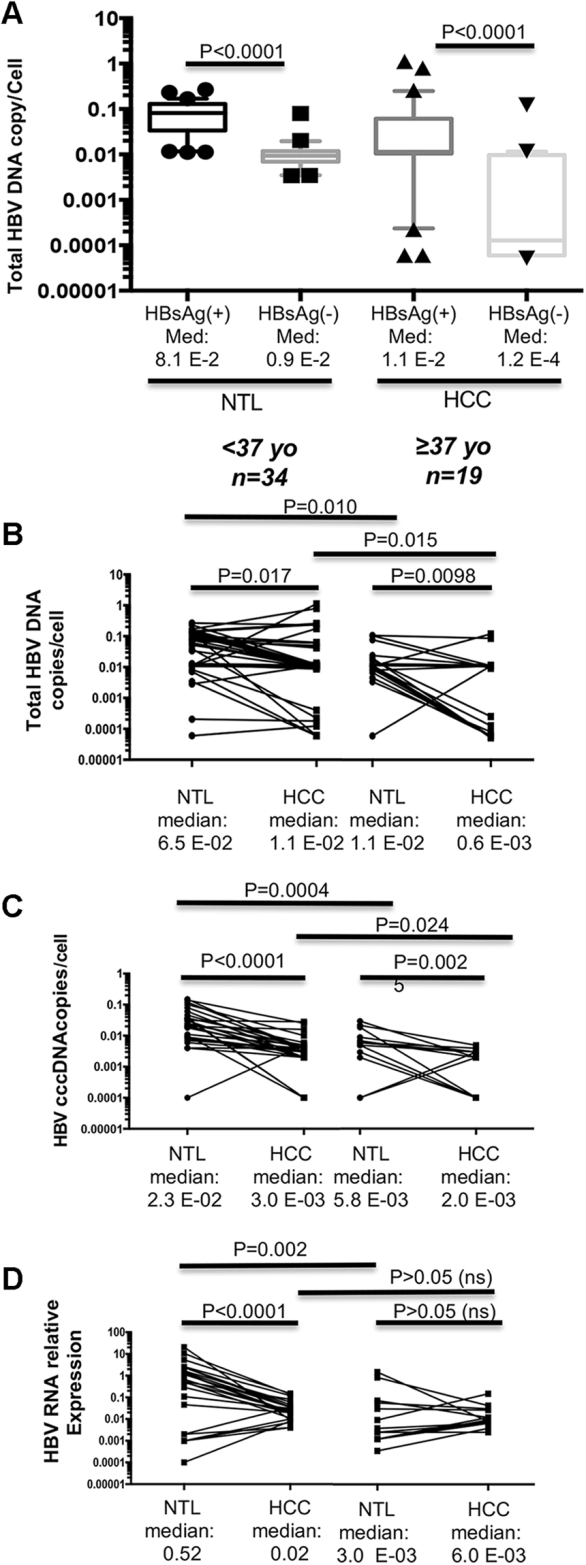
HBV DNA load in liver tumor and non-tumor tissues. (**A**) Box-and-whiskers plots displaying the number of HBV DNA copies per cell as measured by qPCR in HCC and NTL stratified according to HBsAg(+). (**B–D**) Paired dot plots. (**B**) Total HBV DNA copy levels in HCC and corresponding NTL stratified by age [<37 years old (yo) and ≥37 yo]. (**C,D**) Corresponding figures applied to cccDNA (**C**) and HBV RNA expression (**D**). Median cccDNA level in tumor part from older patients is higher than total HBV DNA levels due to the fact that we could not amplify a number of samples with very low levels of cccDNA. The situation generates a mild overestimate of the median. A low default value was given to these samples to include them in statistical analysis. P values are the outcome of a Mann-Whitney U test.

A striking feature was the very low copy number of total HBV genome equivalent [corresponding to covalently closed circular (cccDNA), replication intermediates, and potential HBV integration] per cell with overall median values of 1.0 E-02 and 2.0 E-02 in HCCs and NTLs, respectively (*P* < 0.0001). When comparing the 31 HBsAg(+) patients with the 22 OBI ones, HBV DNA loads were significantly lower both in HCC and in NTL of OBI cases (*P* < 0.0001) (Fig. 2A). We subsequently searched for differences between younger (n = 34) and older (n = 19) patients using median age of the cohort as a threshold (i.e. 37 years old). We observed statistically significant larger amount of total HBV DNA in NTLs than in matched tumor samples (Fig. 2B). Total HBV DNA loads were also higher in HCC/NTL from younger individuals (<37 years old), when compared to older patients (≥37 years old). We then performed qPCR assays targeting cccDNA responsible of viral persistence in liver cells and observed a situation grossly similar to that of total HBV DNA (Fig. 2C). While examining the transcriptional activity of HBV by qPCR, a more active HBV expression was measured in NTLs from younger patients when compared either to corresponding tumor counterparts or to NTLs from older patients (*P* < 0.0001 and *P* = 0.002, respectively) (Fig. 2D). However, transcriptional activity was not significantly different between matched HCC/NTLs from older patients and between HCC specimens of older and younger patients (both *P* > 0.05) indicating that HBV expression decrease is an early phenomenon in cancer cells.

Interestingly, both total HBV DNA and HBV RNA expression tended to be proportional to the amount of cccDNA in NTLs (see Supplementary Fig. S1a). This correlation was not observed in HCC specimens, suggesting that cccDNA is less functional/active in tumor cells (see Supplementary Fig. S1b). Overall, total HBV DNA in NTLs tended to be inversely proportional to age of Peruvian HCC patients (see Supplementary Fig. S1c). In addition, we performed droplet digital PCR (ddPCR) assays that essentially confirmed the initial interpretation of the data (see Supplementary Fig. S2a,b). Taken together, our findings, performed in a context of low HBV DNA burden, demonstrate a progressive loss of HBV DNA functionality (RNA and DNA replication intermediates production) with age in NTLs, coupled with an early loss of HBV DNA activity in tumor cells.

### Hepatitis B virus integration sites

Given the landscape described above, the status of HBV DNA in Peruvian HCC is hardly compatible with paradigmatic liver carcinogenic mechanisms, such as insertional mutagenesis and continuous pro-oncogenic activity of HBx. A “hit-and-run” activity, a non-cell autonomous/micro-environmental process, or some host predisposition are possible alternative explanations for liver tumorigenesis in Peruvian patients. Nevertheless, we assessed HBV DNA integration sites into genome of tumor cells using HBV-*Alu* PCR method^17^. In total, nine integration sites were cloned from seven HBsAg(+) patients and two OBI patients (see Supplementary Table S1). Each integration site was different, with most of them (7/9) located in gene introns. Regarding the genes altered, no common pathway was readily discernible.

### Mutation spectrum of hepatitis B virus DNA

**Figure 3:**
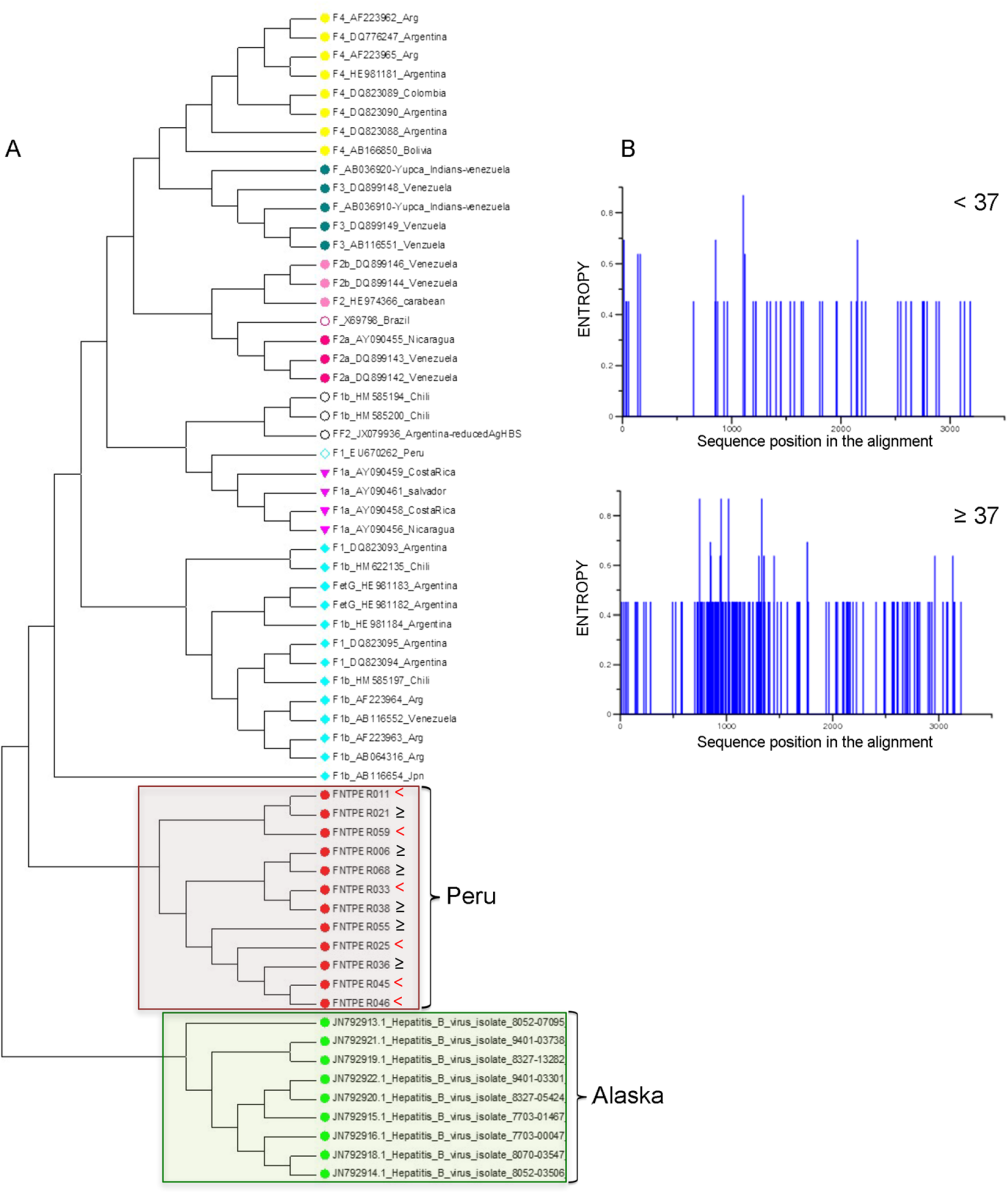
Phylogenic analysis of the full HBV genomes. (**A**) Phylogenic tree construction displaying clustering of various genotype F strains. Red dots correspond to HBV isolates assembled for the present study; all of them were clustered within the sub-genotype F1b. (**B**) Shannon entropy diagram of the 12 HBV isolates from Peruvian patients below (n = 6) (upper panel) and above age 37 (n = 6) (lower panel). Presence of blue lines indicates nucleotide diversity at corresponding position.

To gain more insight into how HBV participate to carcinogenesis in Peruvian HCC, we then decided to sequence full HBV genomes from six younger and six older patients. These HBV DNA genomes were amplified from NTLs, in which they were consistently more abundant than in matched tumors, as mentioned *ut supra*. Overall, phylogenetic analysis clustered all HBV isolates within the sub-genotype F1b (Fig. 3A). Shannon entropy measurement revealed subtle differences between age-based subsets. Indeed, mutation spectra and nucleotide changes in older patients were apparently more polymorphic than those of younger individuals (Fig. 3B).

**Figure 4:**
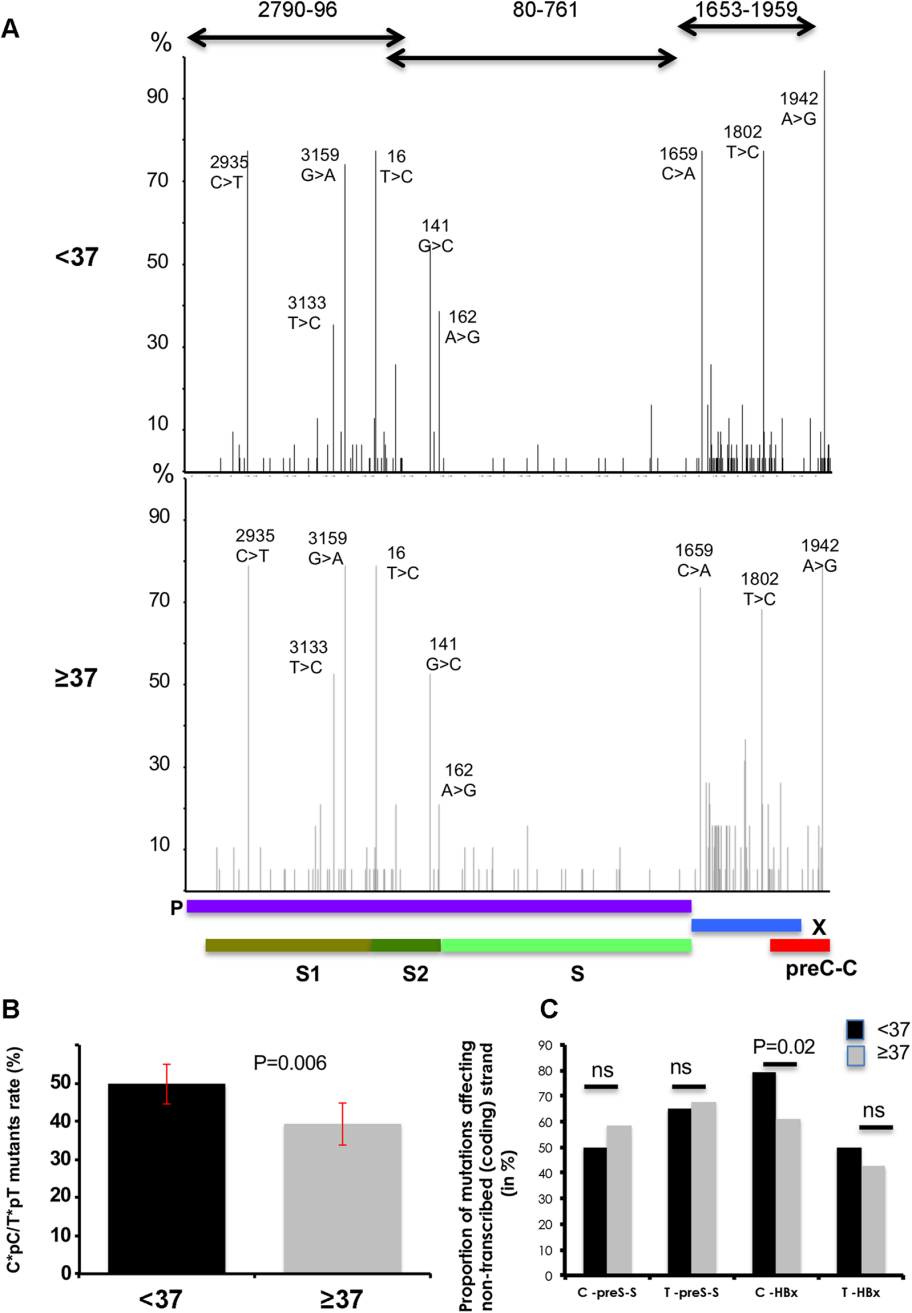
Mutational analysis of HBV. (**A**) Mapping of mutations and percentage of alterations on the 1,492 nucleotides segment resulting from HBV DNA amplification in patients below (n = 34) (upper panel) and above age 37 (n = 19) (lower panel). (**B,C**) Bar charts. (**B**) Proportion of monotonous di-pyrimidines (CpC, TpT) altered in <37 and ≥37 patients. Error bars represent standard deviation. The P value is given by a Chi-square test. (**C**) Cytosine (C) or thymine (T) mutations affecting non-transcribed strand of HBV DNA in <37 and ≥37 patients. The P value is given by a Chi-square test.

We set out thereafter to conduct a more in-depth sequence analysis of HBV strains infecting HCC/NTL from the 53 patients that yielded a PCR product. All isolates belonged to F1b subtype, in accordance to the complete genomes mentioned above. Amplification of three segments corresponding to known variable regions (i.e. PreS-S, HBx, and HBe-HBc) of the HBV genome that represent together a length of 1,492 nucleotides was carried out both in HCCs (when possible due to inherently lower HBV DNA content) and in NTLs (Fig. 4A). As a reference, we used the Peruvian sequence EU670262 published previously by von Meltzer and colleagues^11^. A total 691 genetic variations were detected. Variations of HBV genome were significantly more abundant in NTL than in HCC (557 vs. 371; *P* < 0.0001), presumably because of the higher viral genomic DNA equivalent copy number per cell and a larger pool of mutable targets. Nucleotide mutation spectra of the HBV genome in HCC and NTL were not statistically different (see Supplementary Fig. S3a). Two transitions (T:A > C:G and C:G > T:A), each one accounting for 30–35% of the total mutation load, were predominant. At the positional level, seven patients (14%) were presenting mutants affecting the major hydrophilic region (“a” determinant) with two occultly infected carrying S143W and G145R^18^. Remarkably, non-sense mutations were significantly more frequent in patients with OBI (*P* < 0.0001) (see Supplementary Fig. S3b). By contrast, Pre-S sequence was more often targeted in HBsAg(+) patients (see Supplementary Fig. S3c). We then proceeded with the comparison of sequences obtained from the younger and the older patients. The distribution of mutation targets on the HBV genome was grossly similar in both groups of patients, with HBx being the more heavily altered gene and older patients presenting a trend for a higher proportion of mutations on this particular gene (*P* = 0.07, ns) (see Supplementary Fig. S3d). The only significant differences at the positional level between age subsets concerned the 1,762–1,764 4 AG > TA double mutations which affect basal core promoter, as well as the nucleotide 3,102 in preS1 region (see Supplementary Fig. S3e). Both mutations were significantly more abundant in older than in younger patients (*P* = 0.018). Other mutations considered as clinically important and common in different HBV genotypes, such as nucleotides 1,753 and 1,896, were rare with less than 10%^19^. Mutated triplets tended to be enriched in NTT types (ATT, CTT, GTT, or TTT) in younger patients, as they represented 28.5% of all mutations compared to 22% in older patients (*P* = 0.07, ns) (see Supplementary Fig. S4a). The overall mutational context of nucleotide triplets of HBV genome was marginally different between younger and older patients (*P* = 0.09, ns), suggesting that the mutational processes might be somehow qualitatively different between both groups (see Supplementary Fig. S4b–e).

Interestingly, the analysis of the upstream and downstream contexts at mutated nucleotides clearly showed that monotonous di-pyrimidines (CpC and TpT) and di-purines (ApA and GpG on the minus strand) were significantly more often mutated in younger patients, for which they represent one mutant out of two (*P* = 0.006) (Fig. 4B). In addition, differences in mutation process were apparent when examining for each individual the number and types of mutations. The number of mutations affecting HBV genome was significantly higher in older patients than in younger ones (15.8 ± 1.7 vs. 12.2 ± 0.08; *P* = 0.04); a situation presumably linked to the duration of infection that enables the accumulation of sequence changes. In addition, we observed a mutated strand asymmetry on HBx gene between younger and older patients (Fig. 4C). The number of alterations affecting cytosine, and particularly C > T or C > A, was significantly higher in older patients compared to the younger ones (see Supplementary Fig. S4d–e). Cytosine mutants mostly affected the coding/non-transcribed strand in younger patients, suggesting a transcription-associated DNA damage.

Although we acknowledge that we are presenting a nuanced phenomenon, mutation spectra observed in younger and older Peruvian HCC patients suggest that changes affecting CC/TT dinucleotides or the coding strand of HBx, both found in higher proportion in younger patients, represent early changes affecting HBV genomes in Peruvian HCC patients. The molecular bases of this process, just as its contribution to liver carcinogenesis, are currently unknown. We thus decided to explore the expression of DNA repair genes and viral restriction factors in patient tissues in order to gain molecular insight explaining quantitative or qualitative molecular differences observed in HBV genome.

### Alterations of DNA repair gene expression

The remarkable preservation of liver tissue in Peruvian patients with HCC led us to wonder whether the innate immune response against the HBV could be affected by some kind of constitutive anergy or tolerance. The mutations observed both at HBV DNA (dinucleotides) and host genome (microdeletions) levels associated with the well-known links of HBV replication cycle and oncogenic potential with DNA repair/damage prompt us to investigate the gene expression of key-members of these pathways^14^.

**Figure 5:**
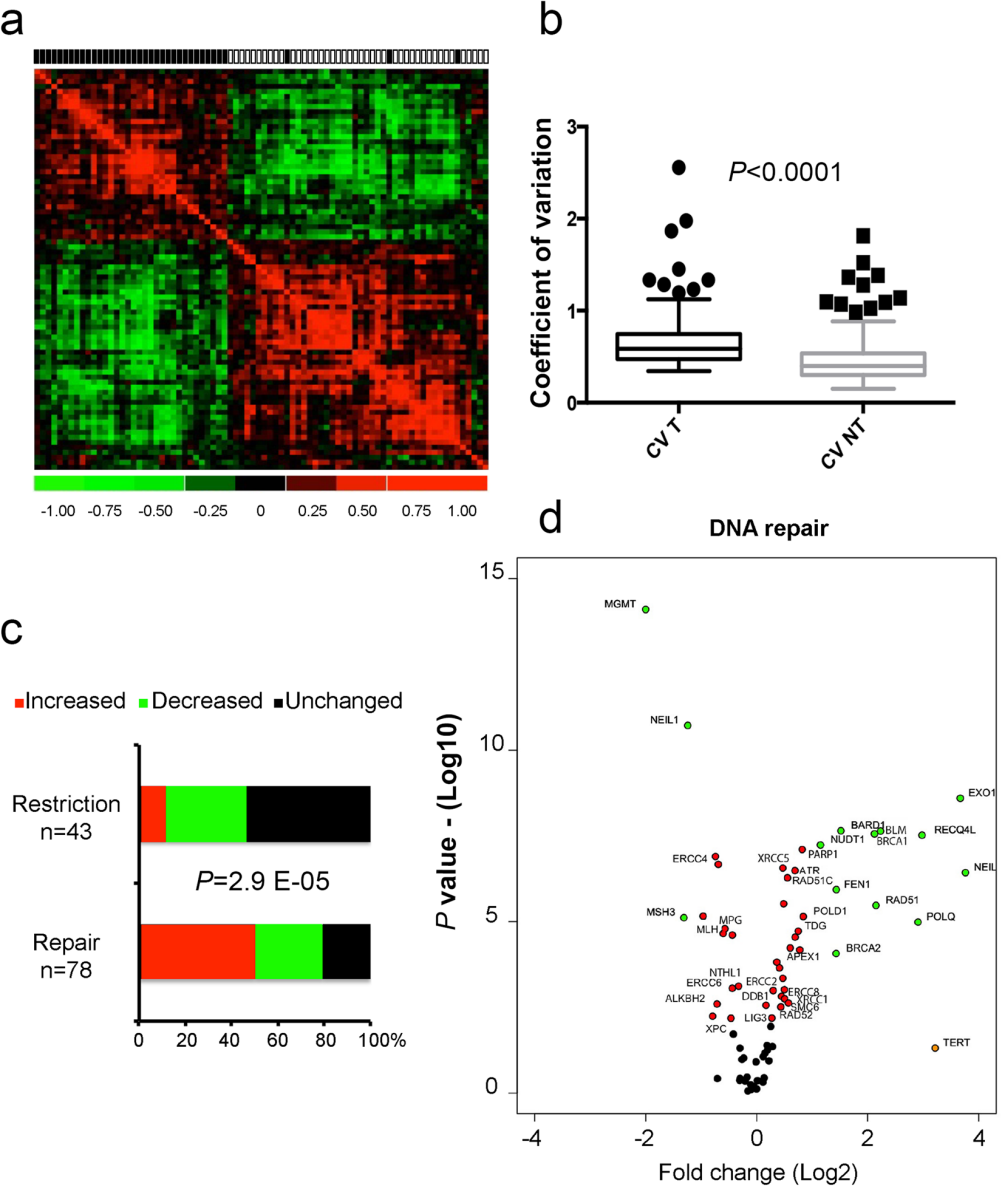
Cellular gene expression analysis. (**a**) Gene-expression correlation matrix of the 121 genes on 40 HCC/NTL matched pairs. Black and white squares above the matrix indicate tumor and non-tumor liver tissues, respectively. (**b**) Box-and-whiskers plots on coefficients of variation of the gene expressions of the 121 genes in the 40 HCC (CV T) and 40 NTL (CV N). P value is given by a Mann-Whitney U test. (**c**) Bar chart of differential expression between the 40 HCC and the 40 corresponding NTL in the viral restriction and DNA repair groups of genes. For each gene, a paired t test or Wilcoxon matched-pairs signed rank test were used as appropriate. (**d**) Volcano plot representing mean fold changes and *P* values of expression affecting the 78 DNA repair genes between the 40 HCC and 40 corresponding NTL. −Log10 of P values were either obtained by Student t test or Mann-Whitney U test depending on the distribution of values as assessed by a *F*-test.

The expression of 121 genes (i.e. 78 involved in DNA repair and 43 in response to viral infections) was assessed using a microfluidic qPCR technique on 40 HCC/NTL matched pairs for which we had high quality RNA (RNA integrity number >7). HCC and NTL gene expressions were drastically different as shown on the correlation matrix (Fig. 5a). An increased variability of gene expression in HCCs compared to NTLs was a consistent outcome of the analysis (coefficient of variation 67.5% vs. 48.2%; *P* < 0.0001) (Fig. 5b). We observed that gene expression variations of DNA repair and viral restriction pathways were strikingly different (*P* = 2.99 E-05). Repair genes were frequently overexpressed in tumors (50%), whereas it was rarely the case for viral restriction genes (5%) that remained unchanged in most cases (53%) (Fig. 5c,d).

**Figure 6:**
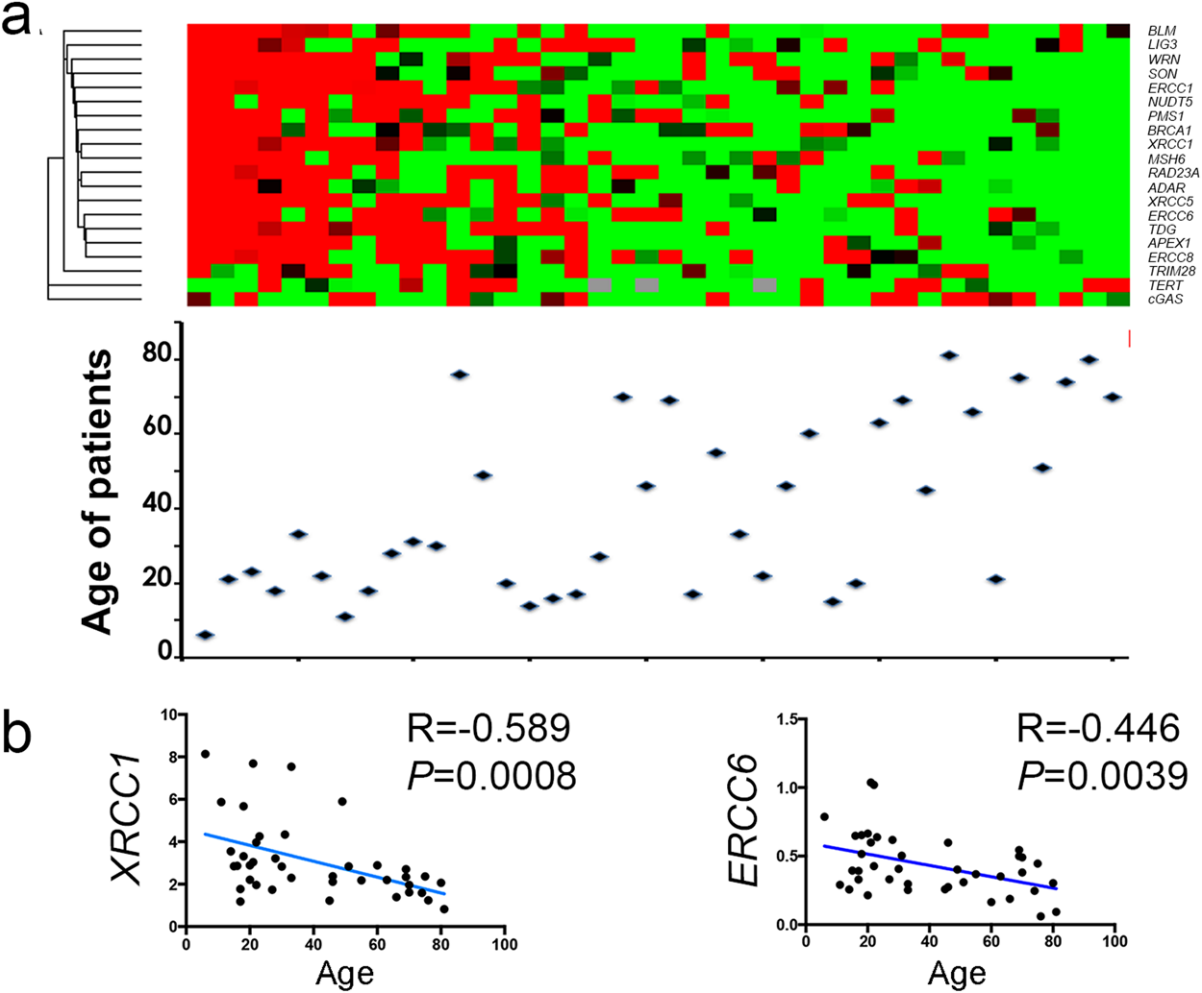
Variation of gene expression according to age. (**a**) Heat map of expression (high = red; low = green) of 20 genes in 40 HCC samples (upper panel). Patients are in columns with their age represented by a diamond (lower panel). (**b**) Correlation plots (Spearman r) of two representative examples of gene expression decrease in tumors cells with patient age (n = 40). Left: X-ray repair cross complementing 1 (*XRCC1*); right: excision repair 6, chromatin remodelling factor (*ERCC6*).

Regarding differences between younger and older patients, we observed that a number of DNA repair gene expressions (n = 16) were significantly decreasing with age (Fig. 6a,b and Supplementary Table S2). This phenomenon was not observed for genes controlling viral lifecycle.

**Figure 7:**
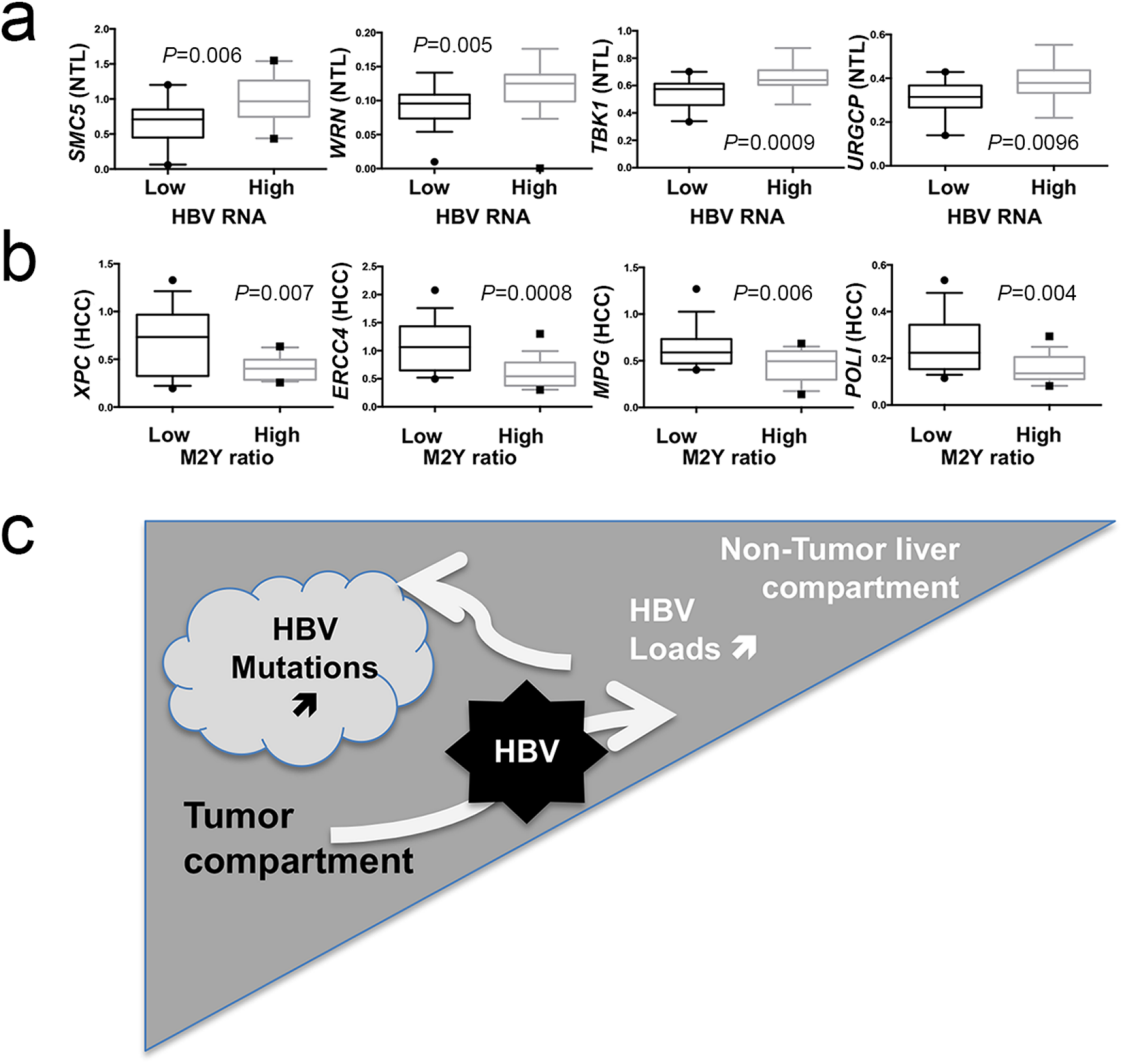
DNA repair gene expression correlates with HBV read-outs. (**a,b**) Box-and-whiskers plots. (**a**) Expression of four DNA repair genes stratified according to the median of HBV RNA expression in NTL (low expression group, n = 18, high expression group, n = 17). From right to left: structural maintenance of chromosome 5 (*SMC5*); Werner syndrome RecQ like helicase (*WRN*); TANK binding kinase 1 (*TBK1*); up regulator of cell proliferation (*URGCP*). (**b**) Expression of four DNA repair genes stratified according to the median proportion of mutated monotonous di-pyrimidines (M2Y = CpC or TpT, low mutated M2Y ratio, n = 15, high ratio, n = 17). From right to left: complex subunit, DNA damage recognition and repair factor (*XPC*); excision repair 4, endonuclease catalytic subunit (*ERCC*); N-methylpurine DNA glycosylase (*MPG*); DNA polymerase iota (*POLI*). Statistical tests for comparison were either Student t test or Mann-Whitney U test depending on the distribution of values as assessed by a *F*-test. (**c**) Graphical abstract proposal for a two-compartment model (i.e. HCC and NTL) explaining viral loads and mutations in Peruvian patients with HCC.

Finally, to gain further insights into variations affecting HBV in Peruvian HCC patients, we looked at relationships between host gene expressions and viral read-outs, such as DNA loads and expression or mutational changes. HBV RNA expression was the most sensitive quantitative read-out. Due to the abundance of presumably more “active” HBV genomes in non-tumor counterparts, host gene expression from NTL rather than from HCC were more frequently correlating with HBV RNA levels (Fig. 7a). Furthermore, when a correlation was found, it was always positive even in the case of viral restriction factors (*URGCP*, *TBK1*, *TLR3*, or *TRIM41*), a configuration possibly triggered by HBV DNA presence itself. Regarding qualitative changes affecting HBV DNA, ratio of mutated di-pyrimidines (or di-purines, M2Y) was the principal feature correlating with a decrease of DNA repair gene expression in tumor tissues. A set of 16 different genes involved in DNA repair were found to be down-regulated in tumors presenting a high rate of CC/TT mutants which was also the case for 10 genes involved in the control the viral life cycle (Fig. 7b). Thus, it appears that decreased DNA repair gene expression in tumor cells is linked with an increased mutation rate affecting HBV genome. Therefore, tumor and non-tumor tissues seem to behave as two functionally different compartments, where neoplastic cells contribute to increase molecular variation and diversity of HBV DNA, whereas NTL is permissive to HBV expression and presumably replenishes viral loads (Fig. 7c).

## Discussion

In Peruvian patients, liver tumorigenesis induced by HBV is characterized by several remarkable features: (i) the low HBV DNA burden both in tumor and non-tumor tissues, (ii) the different mutation spectra affecting HBV DNA in young and older patients and finally (iii) the differential activation of genes involved in various DNA repair pathways according to age.

A salient feature of our work is represented by the systematically low viral burden both in overt and occult forms of infection. It is considered that 5–40% of hepatocytes contain HBV DNA in chronically infected patients with a median value of 1.5 genome equivalent copies per cell^20^. Our data indicate that an almost 100-fold lower abundance of HBV DNA might lead to the development of HCC in a South American context. In addition, the younger age of HBsAg(+) patients and the grossly inverse relationship between HBV DNA copy number in liver tissues and age of patients suggest that overt and occult infections represent sequential segments of the same pathophysiological continuum consisting in a progressive decline of HBV DNA burden and activity (RNA) in liver tissue. The elder age of patients without any detectable HBV DNA leads us to speculate that at least some of them might even represent the ultimate step of a previous HBV infection. Thus, HBV could be at the origin of an overwhelming majority of incident HCC cases in Peru. A predominance of HBV among risk factors of HCC is usually considered a hallmark of countries with high endemicity of persistent infection with HBV^4^. Yet, Peru is usually described as a country of low-to-intermediate endemicity dotted with few hyper-endemic places^8^. The Peruvian situation is also somewhat at odds with the current knowledge that associates high viral loads with a high risk of HCC development^21^. HBV DNA levels in HCC/NTL were measured only at time of HCC diagnosis, and, therefore, we cannot exclude that these patients strongly suppressed HBV replication before developing a liver tumor and that this process promoted subsequent HCC development^22^. This hypothesis seems rather unlikely, as in prospective studies, East Asian patients with the highest risk of HCC were those keeping the highest replication levels until the last follow-up visit^23^. Hence, our data indicate that a high HBV replication level is not a significant determinant of early HCC development in Peruvian patients and that low HBV DNA levels are sufficient to trigger a rapid liver tumorigenesis without the histological changes comprising inflammatory infiltrates, necrosis, fibrosis, or cirrhosis^24^.

At the molecular level, in the presence of much less than one copy per cell, liver tumorigenesis of Peruvian patients is more akin to that observed in OBI; so an indirect carcinogenesis mediated by host response/predisposition rather than a direct and instrumental activity linked to HBV DNA integration, HBx, truncated preS proteins, or even HBV core protein (HBcAg) activity on host gene promoters^25^. The important role of OBI (alone or with hepatitis C virus) in the more advanced forms of liver diseases has been emphasized multiple times, and, despite the uncertainty that surrounds its precise mode of action, it is considered as a *bona fide* risk factor of HCC^26^. In this context, it is important to notice that populations with Americas’ indigenous ancestry from Mexico to Cape Horn are considered to have developed immune defenses leading primarily to mild forms of HBV infection characterized by a pauci-symptomatology^27^. This mostly unapparent form of infection may explain why there is a paucity of reports about OBI and HCC in Latin America, and why OBI are primarily described in HCV-associated cases^28,29^. In Peru, OBI was not previously mentioned, but was presumably responsible for the large subset (30%) of “non-viral” terminal liver diseases (cirrhosis or HCC) observed in the country while ago^28^. However, the issue of OBI has recently emerged as more important than previously thought and mobilized the attention of some Latin American investigators working with blood donors (positive in 0.006–6% of cases), children and adolescent (0.7%), and patients at risk like HIV-positive patients (0.6–49%) and intravenous drug users (2.7–12%)^29^. Another aspect of OBI in Latin America is its apparent enrichment in rural communities living in remote regions, where it was found sometimes in 15.3% of individuals tested^27^. In addition, it has been shown that OBI could be identified as the most plausible participant to liver damage in more than 11% of Mexican children and 8–17% of patients from Yucatan or Western Brazilian Amazon^30^. Overall, the main interest of our data does not reside in the well-admitted observation that a low HBV load or even an OBI might lead, in some circumstances, to HCC, but rather in the observation that it represents a very important form of liver carcinogenesis in a population hitherto considered as only marginally threatened by HBV. This situation is all the more intriguing that HBV genotypes endemic to Americas (i.e. F and H) are usually not recognized as strongly oncogenic in the Latin American epidemiological context^31,32^. This finding led us to hypothesize that another tumorigenic factor synergizes with HBV in Peruvian patients.

In order to find possible fingerprints of this putative cofactor, we analyzed HBV DNA mutation spectrum as a proxy of mutagenic activity. Viral isolates from younger and older Peruvian patients differ according to several features. A salient feature was the higher proportion of mutations affecting monotonous di-pyrimidine (or di-purine on the pair strand) CpC/TpT in younger patients. Di-pyrimidines mutations are considered as the fingerprints for ultraviolet radiations that are for obvious reasons not involved in liver tumorigenesis^33^. The molecular bases of such mutations in a visceral organ remain unknown, although similar alterations were enriched in animal and cellular models using either di-benzyl-nitrosamine or phenanthrene-chrysene derivatives or ion-DNA interacting metals (e.g. copper and iron)^34^. The hypothesis of a role played by phenanthrene-chrysene derivatives is attractive as they were previously shown to target preferentially the non-transcribed DNA strand, a feature recovered in HBx gene of younger Peruvian HCC patients^35^.

Finally, both the differential mutation spectrum for age and the strong repression of HBV genome prompted us to explore expression of cellular genes involved either in DNA repair or in the control of viral cycle. Expression analysis was marked by a conspicuous lack of activation of viral life cycle modulators coupled with a strong activation of DNA repair genes in tumor cells from younger patients. It is known, mostly from animal models, that DNA damage increases with age in the liver, and that this phenomenon is linked to a decreased expression of DNA repair genes^36^. We observed that decreased expression of the DNA repair program occurs primarily in the tumor, but less obviously in NTL. In addition, we showed that HBV DNA level and mutation rates are somehow correlated with DNA repair proficiency both in tumor and non-tumor cells. This situation may explain why HBV DNA is more abundant and less altered in younger patients than in older ones. Finally, the strong expression of DNA repair genes in younger patients raises the issue of the putative stems cells presence in the tumors of younger patients as it is well known that cancer stem cells retain a high capacity to repair DNA damage. Our data suggest that these cells may represent an important component of liver tumor tissue in younger Peruvians. Of course, this hypothesis warrants further investigations, but a recent report from Wang and colleagues indicates that number of DNA repair genes are strongly expressed in HCC from younger Chinese patients when compared to samples from older ones, and that this phenomenon comes along with other stem cell features^37^.

The present study has some limitations that should be corrected in future research. Concerning the sample types analyzed, it would have been useful to analyze plasma samples to compare circulating virus loads with HBV DNA copy number in liver tissues. In addition, the quantitative assessment of circulating HBsAg might represent another interesting piece of information. With regard to gene expression analysis, high-throughput sequencing or microarray analyses will bring unbiased information regarding the pathways involved in liver tumorigenesis of Peruvian patients.

In conclusion, we showed that HBV is very frequently involved in HCC observed in Peru in terms of cases proportion. This situation is to some extents at odds with the notion that Peru is a country with low-to-median endemicity for persistent infection with HBV. In addition, viral burdens are usually very low even in younger patients and occult infections are frequent. Low viral load does not prevent the very early development of HCC as already suggested by Tsai and colleagues in Taiwan^38^. Taken together, these data further delineate the unusual liver disease that affects Peruvian populations. Our investigations should be pushed forward in order to determine whether a particularly stealth form of HBV infection is more frequent in the general population than thought previously and whether the juvenile presentation of HCC is due to some kind of predisposition in populations with a large component of Americas’ indigenous ancestry.

## Methods

### Ethics approval and consent to participate

Written informed consent was provided by participants for their information and samples to be stored in the INEN Department of Cancer Statistics and Epidemiology (for medical charts) and the INEN Department of Pathology (for tissue specimens) and used for research. When the patient was non-adult, a parent provided the informed consent on his behalf. The present study was carried out in strict accordance with the ethical principles contained in the Declaration of Helsinki and was approved by the INEN Human Subjects Committee, protocol numbers #008-2010-CRP-DI/INEN and #113-2014-CIE/INEN.

### Study design and patient selection

Patients with malignant liver neoplasms were managed through the INEN Department of Abdominal Surgery. The patients included in the present study were treated between August 2006 and March 2011 by anatomic liver resection, i.e. systematic removal of the tumor liver segments confined by portal branches to ensure tumor-free margins^39^. Non-tumor livers analyzed were specimens originally adjacent to the tumor. Approximatly 50 mg of both HCC and parent NTL matched pair were harvested from the resected surgical pieces, flash-frozen in liquid nitrogen, and stored at −80 °C (INEN Cancer Research Biobank). After the surgical intervention, pathologists determined tumor size (i.e. longest chord measured), nodule number, and then assessed the type of cancer cells on haematoxylin–eosin-stained sections^40^. Non-tumor liver tissues were treated using the same procedure to determine their degree of inflammation, fibrosis or steatosis. Trained liver pathologists in Lima and Paris confirmed independently HCC diagnosis.

### Nucleic acids extraction

HCC/NTL DNA extraction method has been described elsewhere^14^. DNA concentrations were measured using the Qubit™ dsDNA BR Assay Kit (Invitrogen). RNA extraction from flash-frozen tissues was performed using Tri Reagent^®^ (Sigma-Aldrich) and the Lysin Matrix D homogenization system (MP Biomedicals), according to the manufacturer’s instructions. Purified RNA pools were treated with RNase-free DNase I (Merck). RNA integrity and quantity were assessed using the RNA 6000 Nano LabChip^®^ Kit on a 2100 Bioanalyzer (Agilent Technologies).

### Hepatitis virus DNA detection and mutation analysis

Fifty nanograms of genomic DNA was screened for HBV DNA by PCR, using a nested procedure, on at least three different regions of the viral genome: preS, S, and X-preC. PCR programs included 35 cycles (95 for 1 min; annealing for 1 min; 72 for 1–2 min). Mutations and polymorphisms of HBV DNA were characterized by sequencing according to a Sanger method. Concerning the other hepatitis viruses, 100 ng of cDNA was used to detect both HDV and HCV.

### Hepatitis B virus DNA and RNA quantifications

HBV DNA was quantified by qPCR from 100 ng Qubit-measured genomic DNA extracted from HCC/NTL tissues. Assays were performed in a CFX96™ Real-Time PCR Detection System (Bio-Rad). Total HBV DNA was quantified using the TaqMan^®^ Pathogen Detection Assay Pa03453406_s1 (Thermo Fisher Scientific) adapted for HBV genotype F, whereas amounts of covalently cccDNA were measured as described previously using primers overlapping with the nick of the minus strand (“overgap”)^41^. Amplification specificity of cccDNA was checked by melt-curve analysis. HBV genome equivalent copies were determined on a standard curve generated with known copy numbers of a plasmid containing HBV genome (pFC80). HBV expression analysis was performed by reverse transcriptase qPCR on DNAse I-treated RNA extracted from the same tissues according to a procedure described previously^14^. Genotype F-specific primers located in the HBx-preC region (nt 1,579-1,879) present in all viral transcripts were used.

### Sequencing and phylogeny

Sequences were produced using dideoxy method with the BigDye™ Terminator v3.1 Cycle Sequencing Kit (Applied Biosystems) on each of the nested PCR products after exonuclease I–shrimp alkaline phosphatase treatment (Nucleics). Shannon entropy was calculated using open-access software from the the HIV databases (https://www.hiv.lanl.gov/content/sequence/ENTROPY/entropy_one.html). A phylogenetic tree was computed on genotypes of HBV using Kimura 2 parameter matrix and neighbor joining method on the Molecular Evolutionary Genetics Analysis software version 4.0 (MEGA4).

### Hepatitis B virus-integration site amplification and sequencing

*Alu*-PCR amplifications of integrated HBV sequences were performed as described by Minami and colleagues^17^. PCR products were cloned in a PCRII^®^ TOPO^®^ vector using the TOPO^®^ TA Cloning^®^ Kit (Invitrogen) and transformed in One Shot™ TOP10 Chemically Competent *E. coli* (Invitrogen) for subsequent sequencing according to a Sanger method.

### Gene expression analysis

Gene expression analysis of 40 HCC/NTL as well four normal liver tissues was performed using a BioMark HD™ Real-Time PCR System (Fluidigm), according to manufacturer’s instructions. Briefly, pre-amplification of 200 ng cDNA was performed by pooling all primers at a final concentration of 0.5 µM. Amplifications were carried out at 95 °C for 10 min, followed by 10 PCR cycles at 95 °C (15 s) and 60 °C (4 min). A final 1x concentration of SsoFast™ EvaGreen® Supermix with Low ROX (Bio-Rad) was added to each pre-amplified cDNA and 5 µM of each primer pairs were loaded on 96.96 Dynamic Array^TM^ IFC (Fluidigm). Amplifications were carried out at 95 °C for 1 min, followed by 30 PCR cycles at 96 °C (5 s) and 60 °C (20 s) on the Biomark System. Data was analyzed using the three reference genes [hydroxymethylbilane synthase (*HMBS*), lipase maturation factor 2 (*LMF2*), and tripartite motif containing 44 (*TRIM44*),]. The relative quantitation and expression fold were determined by the ΔCq and the ΔΔCq methods respectively. QPCR assays were performed according to the Minimum Information for Publication of Quantitative Real-Time PCR Experiments guidelines and Real-Time PCR Data Markup Language structured and universal data standard. QPCR assays were performed in triplicate for each sample. Non-supervised clustering was realized using DChip analyzer (http://www.dchip.org/).

### Droplet digital PCR

DdPCRs were performed on QX100™ Droplet Digital™ PCR System (Bio-Rad) using the TaqMan^®^ Pathogen Detection Assay Pa03453406_s1 and the Human TaqMan^®^ Copy Number Reference Assay (Thermo Fisher Scientific) as a reference. Reaction mixtures consisted of 10 µl of ddPCR™ Supermix (Bio-Rad), 1x primers, and 100 ng of total DNA in a final volume of 20 µl. About 70 µl of Droplet Generation Oil (Bio-Rad) was used to create an emulsion of monodispersed droplets using QX100™ Droplet Generator (Bio-Rad), and emulsified samples were transferred to a 96-well PCR plate. Duplex PCRs were performed on a CM1000 Touch™ Thermal Cycler (Bio-Rad) with the following thermal cycling protocol: denaturation at 95 °C for 10 min, followed by 40 cycles at 94 °C for 30 s with a 2.5 °C/sec ramp rate, 59 °C for 1 min with a 2.5 °C/sec ramp rate, 98 °C for 5 min, and hold at 4 °C. After PCR, 96-well plates were scanned in a QX100 Droplet Reader (Bio-Rad). The data was analyzed using QuantaSoft™ software (Bio-Rad) with autoanalysis settings for duplex experiment.

### Statistical analysis

All statistical analyses were performed using Prism Mac 6.0 d software (GraphPad). Data were presented as mean ± standard deviation or as median value as appropriate. Prevalence is given as percentages. All tests were two-sided. The level of significance was set as *P* < 0.05.

### Data availability

All data generated or analyzed during this study are either included in this published article or its Supplementary Information file. Sequences have been submitted to the European Bioinformatics Institute (EBI) and are accessible through PRJEB21100 project accession number (https://www.ebi.ac.uk/ena/data/view/PRJEB21100).

## Electronic supplementary material

Supplementary Information

Supplementary_Table_S2_SREP-18-10360A
