# Supplementary material for: Early-onset liver cancer in South America associates with low hepatitis B virus DNA burden
Source: Sci Rep. 2018 Aug 13;8:12031. doi: 10.1038/s41598-018-30229-8 (PMC6089985; doi:10.1038/s41598-018-30229-8)
Supplement: Supplementary file 1 — Supplementary Information [file 41598_2018_30229_MOESM1_ESM.docx]

**Early-onset liver cancer in South America associates with low hepatitis B virus DNA burden.**

**Ms_SREP-18-10360A**

Agnès Marchio, Juan Pablo Cerapio, Eloy Ruiz, Luis Cano, Sandro Casavilca, Benoît Terris, Eric Deharo, Anne Dejean, Stéphane Bertani, Pascal Pineau

**
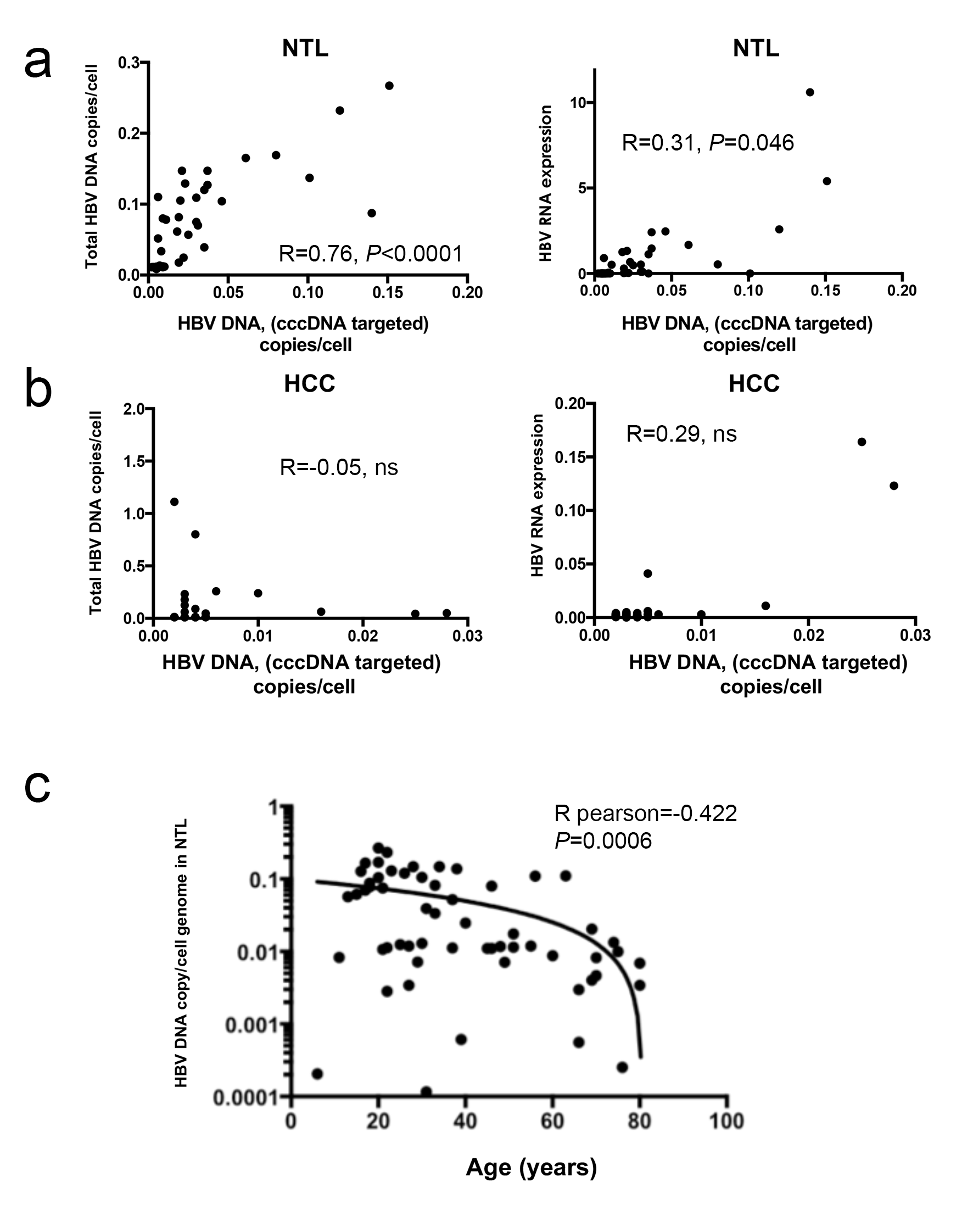
**

**Supplementary Figure S1. HBV activity depends primarily on non-tumor liver tissue.** (**a-c**) Correlation plots. (**a**) HBV cccDNA levels vs. total HBV DNA levels (left, n=30) and HBV RNA expression (right,n=25) in NTLs. (**b**) HBV cccDNA levels vs. total HBV DNA levels (left,n=15) and HBV RNA expression (right, n=15) in HCCs. (**c**) Total HBV DNA vs. age (n=53).


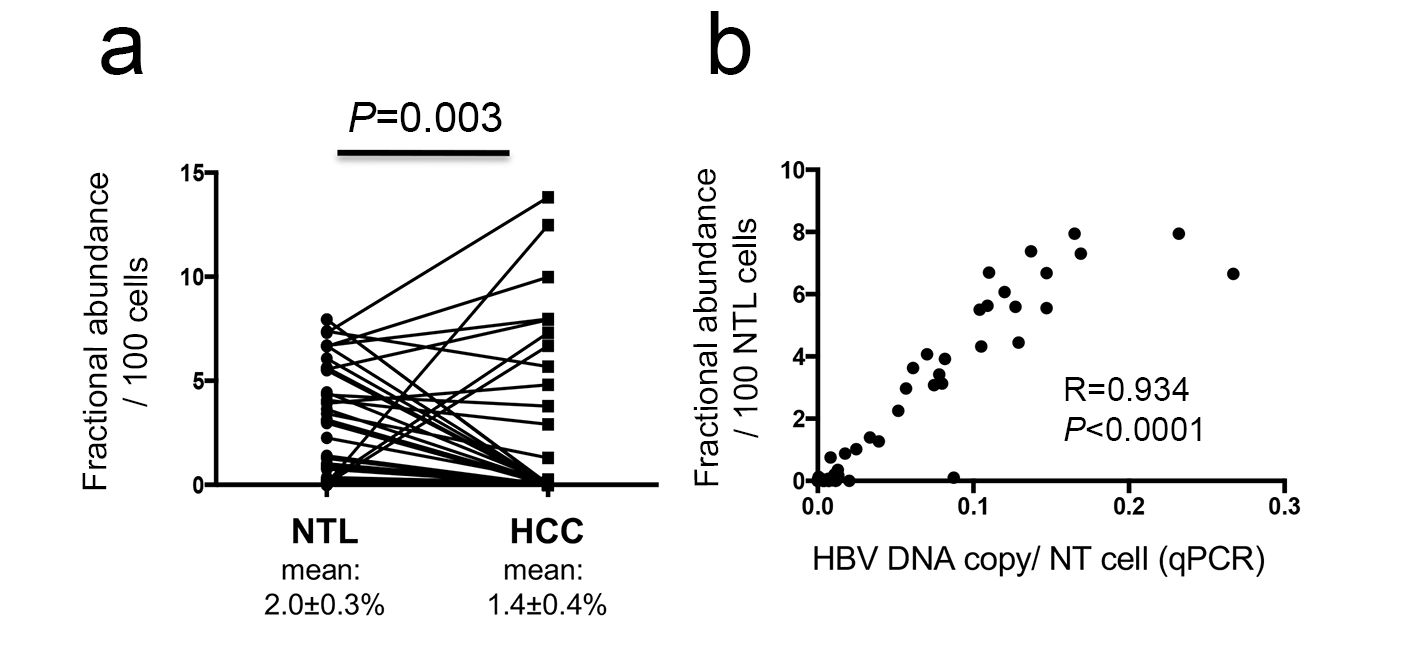


**Supplementary Figure S2. Validation of qPCR with digital droplet PCR.** (**a**) Paired dot plot on abundance of HBV DNA as measured in HCC/NTLs using ddPCR assays (n=31). (**b**) Correlation plot of standard qPCR measurements of total HBV DNA in NTLs vs. ddPCR outcomes on the same target (n=31).

**Supplementary Table S1. HBV DNA integration sites isolated from HCC DNA of Peruvian patients.**

| Tumor ID | Sex/Age | Serology | Cytoband | Gene symbol | Location | Full name |
| --- | --- | --- | --- | --- | --- | --- |
| HCCPER1 | M25 | HBs(+) | 5q14 | *ATP6AP1L-TMEM167A* | intergenic | ATPase, H+ transporting, lysosomal accessory protein-like/Transmembrane protein |
| HCCPER4 | M70 | HBs(-) | 2q35 | *STK11IP* | intron 3 | Serine/threonine kinase 11 interacting protein |
| HCCPER11 | M17 | HBs(+) | 7p21 | *AGMO* | intron 5 | Alkylglycerol monooxygenase |
| HCCPER12 | F23 | HBs(+) | 9p22 | *NFIB* | intron 1 | Nuclear factor I/B |
| HCCPER45 | F13 | HBs(+) | 10p15 | *SFMBT2-ITIH5* | intergenic | Scm-like with four mbt domains/Inter-alpha-trypsin inhibitor heavy chain family |
| HCCPER65 | F81 | HBs(+) | 18q21 | *ZNF352* | intron 3 | Zinc finger protein |
| HCCPER70 | M64 | HBs(-) | Not determined | *repetitive* | Not determined | Not determined |
| HCCPER73 | M22 | HBs(+) | 6p21 | *MRPS10* | intron 1 | Mitochondrial ribosomal protein S10 |
| HCCPER76 | M31 | HBs(+) | 15q26 | *CRTC3* | intron 1 | CREB regulated transcription coactivator 3 |

**
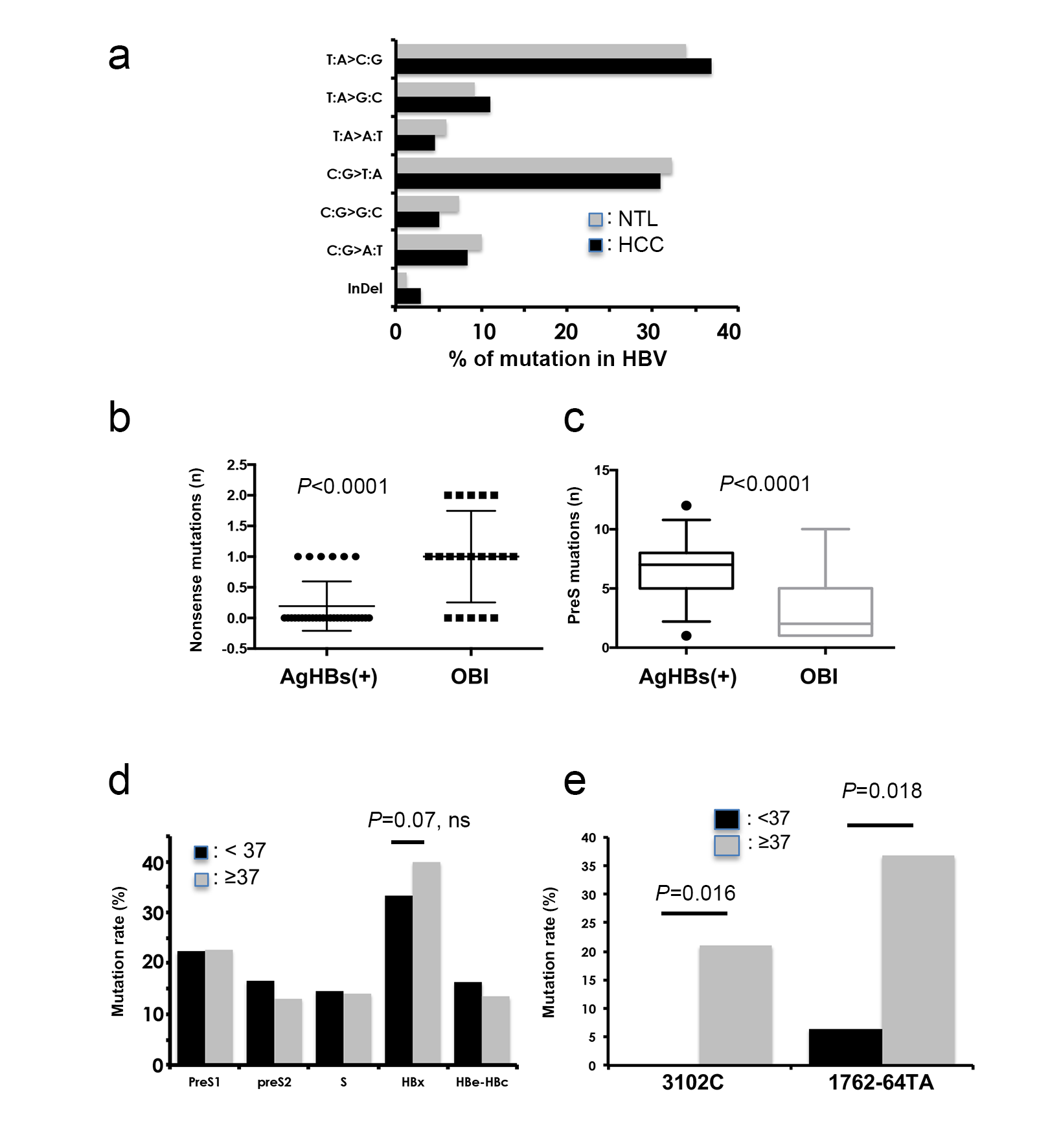
**

**Supplementary Figure S3. Mutation spectrum of HBV DNA.** (**a**) Bar chart representing the proportion of nucleotide mutation classes in HCC (black, n=53) and NTL (grey, n=53). (**b,c**) Box-and-whiskers plots. (**b**) Nonsense mutations are more abundant in patients with occult B infection (OBI, n=22) than in patients with overt infection (n=31). (**c**) Pre-S mutants are more abundant in HBsAg(+) patients (n=31) than in patients with OBI (n=22). (**d,e**) Bar charts. (**d**) Mutation distribution throughout HBV DNA in younger (black, n=34) and older patients (grey, n=19). (**e**) Differential mutation rate on common hotspots of variations according to age of patients (<37, n=34, and >37, n=19).


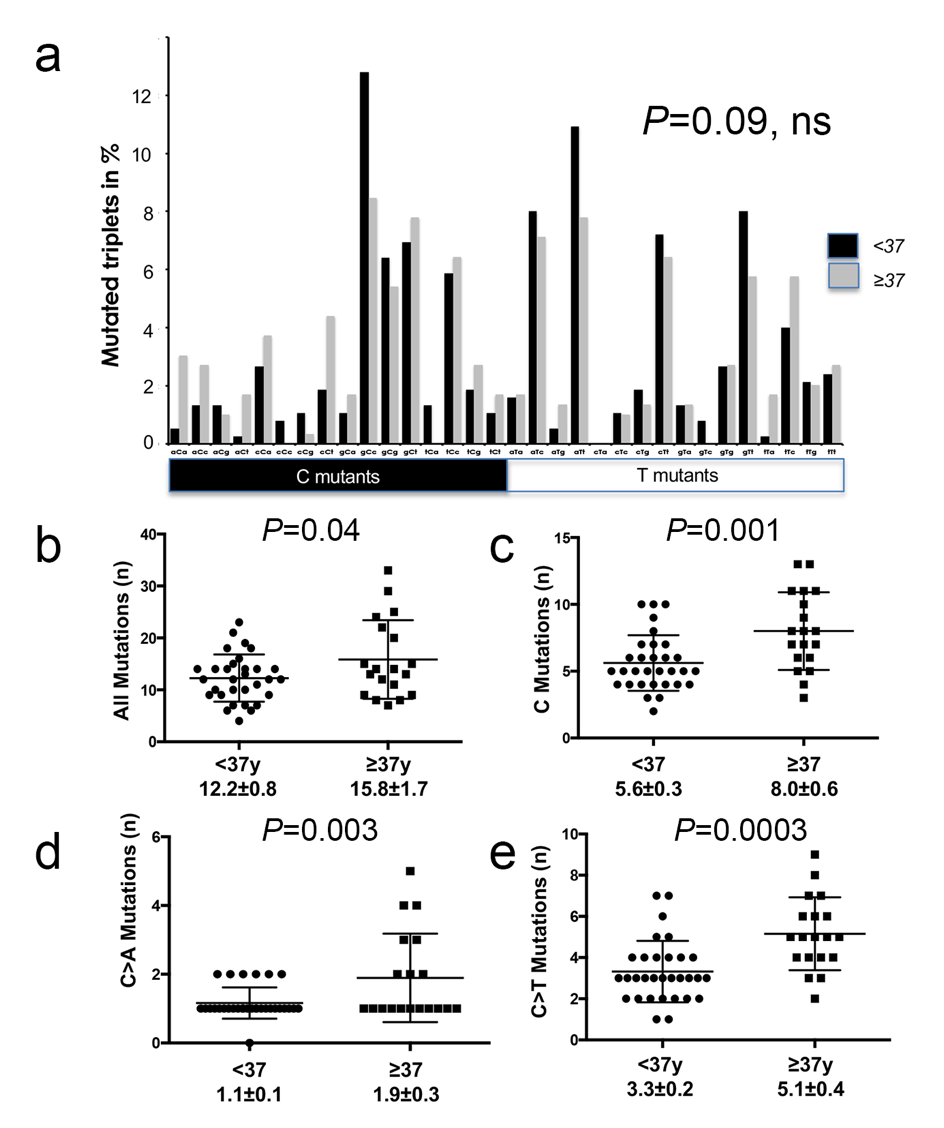


**Supplementary Figure S4. Differential mutation spectrum of HBV DNA according to patient age.** (**a**) Bar chart displaying mutated triplets <37 (black, n=31) and ≥37 (grey, n=19) patients. ns, not significant. (**b,e**) Box-and-whiskers plots. (**b**) Total number of mutation affecting HBV DNA in <37 (n=31) and ≥37 patients (n=19). (**c**) Mutations affecting cytosine in <37 and ≥37 patients. (**d**) C>A mutations affecting HBV DNA in <37 and ≥37 patients. (**e**) C>T mutations affecting HBV DNA in <37 and ≥37 patients.
